# Impact of the Initial Phase Composition of Alloys on the Effects Manifested by Yield Sites That Occur on Sheet Aluminum Alloys Subjected to Impact-Oscillatory Loading

**DOI:** 10.3390/ma16010249

**Published:** 2022-12-27

**Authors:** Mykola Chausov, Andrii Pylypenko, Pavlo Maruschak, Vira Zasimchuk, Janette Brezinová, Jakub Brezina, Ján Viňáš

**Affiliations:** 1 Department of Mechanics, National University of Life and Environmental Sciences of Ukraine, Heroiv Oborony Str. 15, 03041 Kyiv, Ukraine; 2 Department of Industrial Automation, Ternopil National Ivan Puluj Technical University, Rus’ka Str. 56, 46001 Ternopil, Ukraine; 3 G.V. Kurdyumov Institute for Metal Physics, NAS of Ukraine, 36 Academician Vernadsky Boulevard, 03142 Kyiv, Ukraine; 4 Department of Technology, Materials and Computer Supported Production, Faculty of Mechanical Engineering, Technical University of Košice, Mäsiarska 74, 04001 Košice, Slovakia

**Keywords:** dynamic non-equilibrium processes, aluminum alloys, static tensioning, alloy plasticization

## Abstract

The impact of the initial phase composition of alloys was evaluated, in particular, the content of Cu, Mn, and Mg in aluminum alloys D16ChATW, 2024-T351 and aluminum alloy T, which in its physical and mechanical characteristics is close to alloy 6013. The impact was evaluated on the effects manifested by yield sites that occur on aluminum alloys that were subject to the dynamic non-equilibrium processes (DNPs) at the expense of impact-oscillatory loading of different intensities under conditions of static tensioning, The one-time DNP, to which the investigated aluminum alloys were subjected at the pre-set levels of elastic strain followed by static tensioning, was found to cause yield sites formation. This is due to self-organization of the alloy structure, which contributes to alloy plasticization. The initial phase alloys composition impact on the yield sites, which occurs when impulse energy of a different intensity is applied to the alloys, was analyzed. The specimens from the aluminum alloys undergoing DNPs of the same level were compared. This made it possible to conclude that alloys D16ChATW and 2024-T351, which have a higher content of Cu, Mn, and Mg, have longer yield sites upon subsequent static tensioning. On the basis of the experimental results, in particular, physical studies, the authors derived a physical and mathematical model of the yield sites that appear after DNPs.

## 1. Introduction

Under certain temperature- and velocity-related test conditions, polycrystalline materials exhibit an abnormally high (a hundred-fold and even a thousand-fold) elongation at relatively low yield stresses [1,2]. This ability is referred to as superplasticity [3,4]. The temperature range, within which the structural superplasticity exists, is broad enough [5,6]. It may vary for different metals and alloys, ranging from the recrystallization temperature 0.4T_melt_ to temperatures that approximate to melting point [6,7]. The vast majority of materials and alloys known to date are in the optimal strain rate range that corresponds to structural superplasticity, that is, 10^−2^–10^−4^ s^−1^ [8,9]. Normally, super-plasticity occurs in metals and alloys with particularly fine or ultrafine grains, the grain size being less than 10 μm. The yield stress inherent in superplastic deformation is significantly lower than the yield point, this being the major peculiarity of this strain state [10,11]. Superplastic materials are practically incapable of strain hardening (n → 0), and a “neck” is not formed [12,13]. Boundary grain sliding, diffusional creep and intra-granular sliding were found to be the main mechanisms of superplastic deformation (SPD) [14,15].

The material viscous flow nature is characterized by superplasticity and quasi-super-plasticity (similar to the utmost plastic state), which is associated with the diffusional mass transfer along and around grain boundaries [16,17]. From a rheological perspective, viscous flow may be inherent in the deformed material quasi-liquid phase [18,19,20]. At the same time, rheological approaches that describe the deformation process in a wide range of velocities suggest the existence of several deformation mechanisms acting at different stages [21,22,23].

The possibility of the quasi-liquid phase being formed at grain boundaries between fine-grained materials and alloys under superplastic deformation has been proven [24,25] via the thermodynamic approach. This phenomenon will draw much attention, as it explains the manifestations of quasi-super-plasticity under impulse loading of sheet materials [26,27]. The main mechanisms that explain the state of super-plasticity are based on the assumption of liquid phase areas that exist at grain boundaries, or liquid boundaries at high temperatures, which contribute to grain sliding at the boundaries. This assumption was confirmed by the experiment [24,25,26,27].

There must be a number of additional factors that support the assumption of a liquid phase formed at grain boundaries under high-speed super-plasticity. One of them is the temperature at which high-speed super-plasticity occurs, that is, from 0.95 T_melt_ to T_melt_. In addition, a significant adiabatic heating that results from high strain rates is an added factor that supports the above assumption [2]. Therefore, the presence of a viscous-liquid structure in the material is crucial for the analysis of various types of super-plasticity and its physical and mechanical nature.

The authors had found earlier [28,29] that the sheet aluminum alloys D16ChATW and 2024-T351 subjected to additional impulse loads of different intensities at room temperature under impact-oscillatory loading followed by static tensioning at the same temperature, start exhibiting quasi-super-plasticity properties, with a sharp yield point and yield sites of different lengths appearing. The authors attributed this effect to a thin-banded (amorphous-like) dissipative structure that occurs in the material volume at different scale levels upon dynamic non-equilibrium processes (DNPs). It was experimentally recorded using a direct physical method of transmission electron microscopy (TEM) [30,31].

This suggests that under significant strain rate variations, aluminum alloys are prone to self-organization that entails short-term structural rearrangements even at room temperature. An amorphous-like (viscous-liquid) structure featuring hydrodynamic flow channels [31] is formed here, with yield sites of different lengths appearing upon further static tensioning.

This research is aimed at finding conditions under which the structural quasi-super-plasticity (similar to the superplastic state) occurs upon one-time and two-time additional impulse loads applied to sheet aluminum alloys with different initial phase compositions, in particular, a different percentage of Cu, Mn, and Mg, under impact-oscillatory loading at room temperature. Deriving a physical and mathematical model of this process is also important.

## 2. Materials and Methods

According to Table 1 the main difference in chemical composition of the investigated aluminum alloys lies in a significant difference between the total content of Cu, Mn and Mg (in %). Moreover, the maximum content of these elements is in alloy 2024-T351 (6.9%); a slightly lower content is in alloy D16ChATW (6.43%), and a significantly lower one is in alloy T (0.879%).

The mechanical testing technique was implemented based on a modified ZD-100Pu hydraulic installation for static testing and is described in detail in [32]. The proposed technique’s main idea consists of high-speed material tensioning with the imposition of high frequency (1–2 kHz) oscillatory process, which corresponds to the testing machine natural frequency. Structurally, this is achieved by an inner contour introduced into the testing machine in addition to the outer contour (testing machine-loaded frame). The inner contour is the simplest statically indeterminate structure in the form of three parallel elements loaded simultaneously—the central specimen and two satellites (brittle samples) of different cross-sections made of the hardened steels 65 G or U8–U12. When this structure is tensioned, the satellites are destroyed, and the energy is introduced into the specimen under study in a pulsed manner. Satellites may get involved in the operation at any stage of preliminary static tension, making it possible to study the impulse introduction energy effect on mechanical properties degradation by the material being damaged in the static tension process. By changing the initial satellites diameter, it is possible to control the impulse intensity introduction of the force energy into the material.

The value of ε_imp_ under DNPs was controlled by the optical method [33]. Subsequently, stress-strain curves of all the alloys subjected to the repeated static tensioning were recorded. Structural changes in the alloys after DNPs were examined on the Scanning Electron Microscope JSM-6610nx (Jeol. Ltd., Tokyo, Japan) and Transmission Electronic Microscope JEOL–200CX (Jeol. Ltd., Tokyo, Japan).

**Figure 1:**
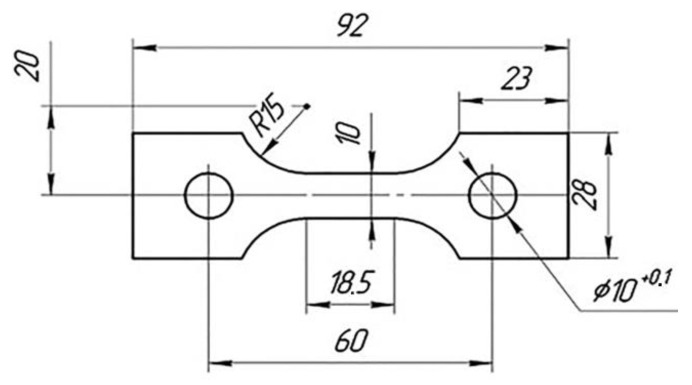
Test specimen (dimensions indicated in mm).

Mechanical tests were performed on specimens (Figure 1) made of D16ChATW, 2024-T351 and T sheet industrial aluminium alloys with a thickness of 3 mm at a room temperature. The strain measurement base was 16 mm. In this case, a standard 0.5 accuracy class extensometer manufactured at the Antonov aircraft production plant, Ukraine, was used for strain measurements.

Mechanical properties of the investigated alloys were assessed in accordance with ISO 6892:1984. The chemical composition assessment of alloys was carried out on an X-ray fluorescence spectrometer VRA-20 (Carl Zeiss, Germany, Jena). The chemical composition of investigated aluminium alloys and mechanical properties there are shown in Table 1 and Table 2.

It should be noted (see Table 2) that the main differences in the chemical composition of the studied aluminum alloys lie in a significant difference in the total amount of Cu, Mn chemical elements and Mg (in %). Moreover, the maximum content of these elements is in the 2024-T351 alloy (6.9%), slightly less in the D16ChATW alloy (6.43%) and significantly less in the T alloy (0.879%).

## 3. Results and Discussions

### 3.1. Plasticization of Aluminum Alloys Subjected to DNPs on the Elastic Part of Stress–Strain Diagrams. Mechanical Test Results

**Figure 2:**
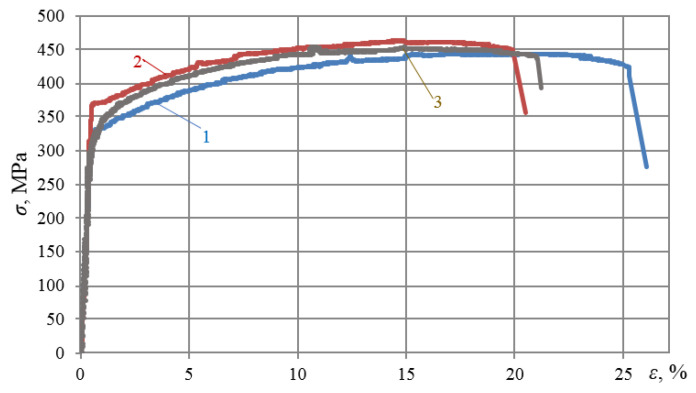
Stress–strain curves of aluminum alloys at room temperature: 1—D16ChATW; 2—2024-T351; 3—T alloy.

Figure 2 shows stress–strain diagrams of alloys in their initial state at room temperature. As can be seen, chemical composition affects the mechanical properties of alloys. In particular, the yield strength *σ*_ys_ varies in the range of 322–372 MPa, the ultimate strength (*σ*_us_) varies in the range of 448–462 MPa, and the maximum strain varies in the range of 21.1–25.2%.

For a detailed DNP effect analysis, the dynamic strain increments (ε_imp_) of specimens in the process of energy impulse introduction were set as [28,29]. The DNP intensity impact on the effects manifested by yield sites that occur in the alloy was studied using a special technique that takes into account the ε_imp_ parameter. A series of specimens from the aluminum alloys 2024-T351, D16ChATW and T was tensioned sequentially to the elastic strain level of (0.21–0.60%) and subjected to force impulse loads in the range of 42–56 kN, and was subsequently fully unloaded. To each alloy studied, nine additional impulse loads were applied under DNPs of different intensity. The DNP course was monitored by the dynamic impulse strain level—ε_imp_. The DNP realization with a slight initial deformation was selected intentionally to reduce the material damage impact on the process nature. The authors had found earlier that at low ε_imp_ values, the alloy structure’s self-organization only begins to manifest itself upon DNPs [28,29]. Notably, in the ε_imp_ range of 3.5–4.5%, the fatigue life of the aluminum alloys D16ChATW and 2024-T351 is significantly improved under further cyclic deformation [28,29]. Therefore, in this study, the ε_imp_ range of 4.26–9.38% was used for alloy D16ChATW, ε_imp_ range of 3.72–10.0% was used for alloy 2024-T351, and ε_imp_ range of 3.12–9.69% was used for T alloy.

**Figure 3:**
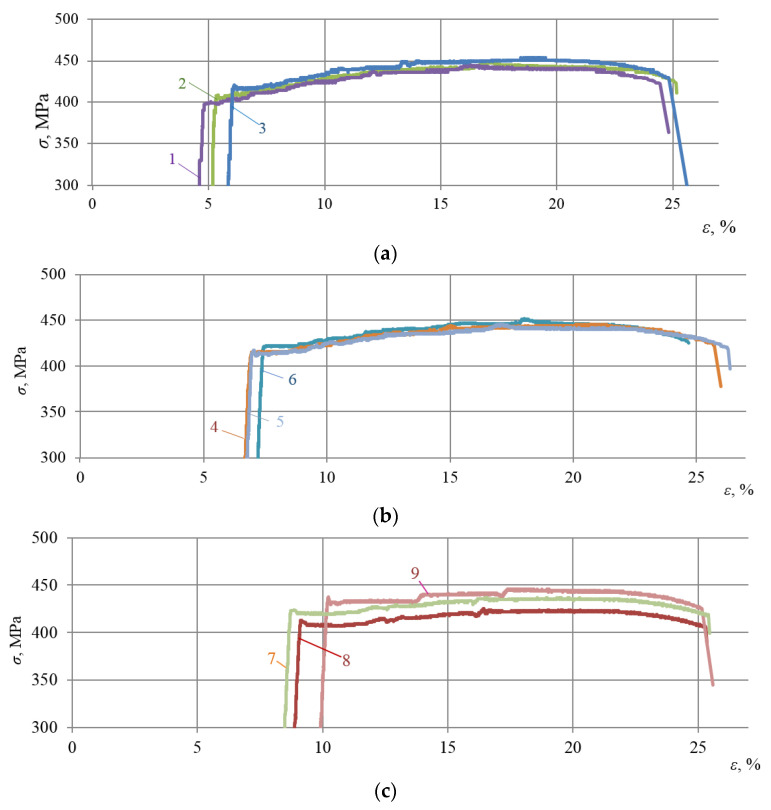
Deformation diagrams of the D16ChATW alloy during static stretching after DNP of different intensities: (**a**)—1—ε_imp_ = 4.26%; 2—ε_imp_ = 4.71%; 3—ε_imp_ = 5.40%; (**b**)—4—ε_imp_ = 6.21%; 5—ε_imp_ = 6.27%; 6—ε_imp_ = 6.69%; (**c**)—7—ε_imp_ = 8.0%; 8—ε_imp_ = 8.39%; 9—ε_imp_ = 9.38%.

**Figure 4:**
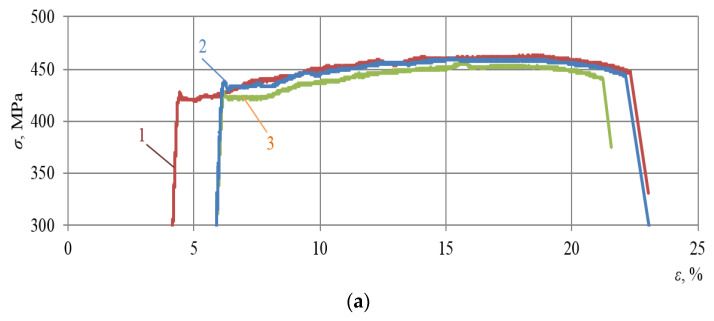

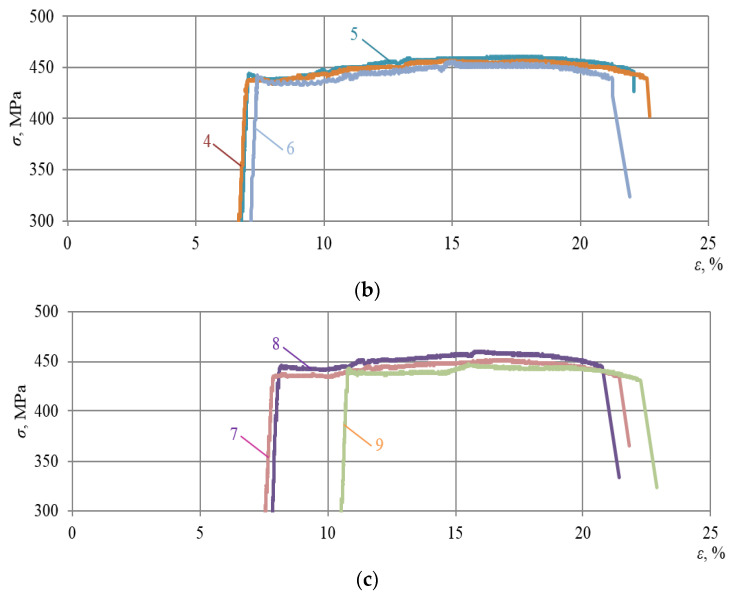
Deformation diagrams of the 2024-T351 alloy during static stretching after DNP of different intensities: (**a**)—1—ε_imp_ = 3.72%; 2—ε_imp_ = 5.43%; 3—ε_imp_ = 5.44%; (**b**)—4—ε_imp_ = 6.31%; 5—ε_imp_ = 6.32%; 6—ε_imp_ = 6.67%; (**c**)—7—ε_imp_ = 7.14%; 8—ε_imp_ = 7.38%; 9—ε_imp_ = 10.0%.

**Figure 5:**
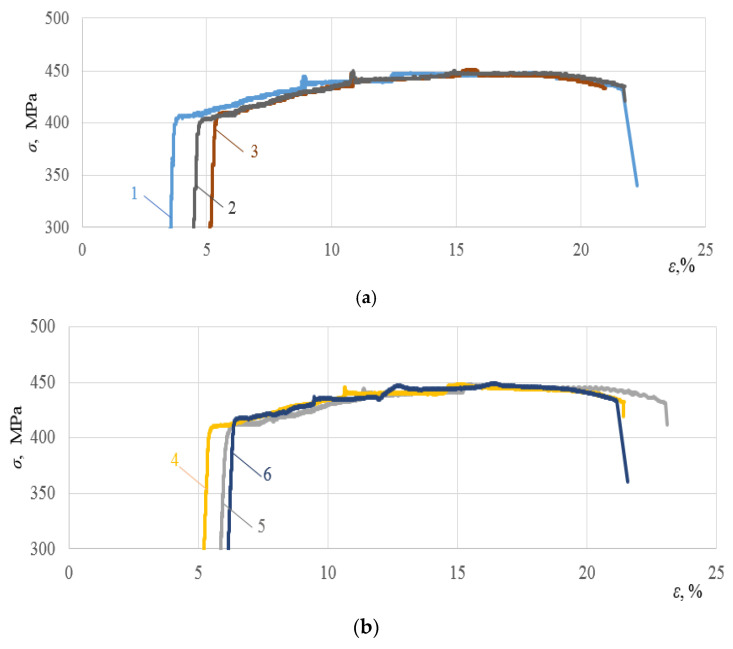

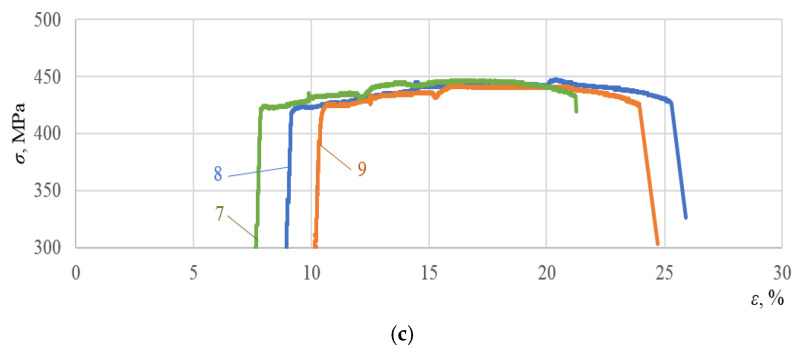
Deformation diagrams of the T alloy during static stretching after DNP of different intensities:: (**a**)—1—ε_imp_ = 3.12%; 2—ε_imp_ = 4.06%; 3—ε_imp_ = 4.69%; (**b**)—4—ε_imp_ = 4.75%; 5—ε_imp_ = 5.37%; 6—ε_imp_ = 5.75%; (**c**)—7—ε_imp_ = 7.25%; 8—ε_imp_ = 8.44%; 9—ε_imp_ = 9.69%.

Subsequently, stress–strain curves of all the alloys subjected to repeated static tensioning were recorded. Test results are presented in Figure 3, Figure 4 and Figure 5.

The analysis of Figure 3, Figure 4 and Figure 5 allows us to draw a number of important conclusions. Firstly, with the selected minimum values of ε_imp_, the yield sites only begin appearing upon subsequent static tensioning for almost all the alloys studied (see Figure 3a, Figure 4a and Figure 5a). Moreover, in this case, the difference between the physical and mechanical characteristics inherent in the formation of these sites was already revealed. Thus, for instance, in contrast to alloy T, alloys D16ChATW and 2024-T351 exhibited a sharp yield point, which was detected experimentally. Secondly, all the alloys studied were characterized by longer yield sites when the DNP intensity was increased according to the ε_imp_ parameter. Thirdly, alloy T with a much lower Cu, Mn, and Mg content exhibits a significantly more difficult occurrence of yield sites compared to alloys D16ChATW and 2024-T351, which have a higher content of these elements.

**Figure 6:**
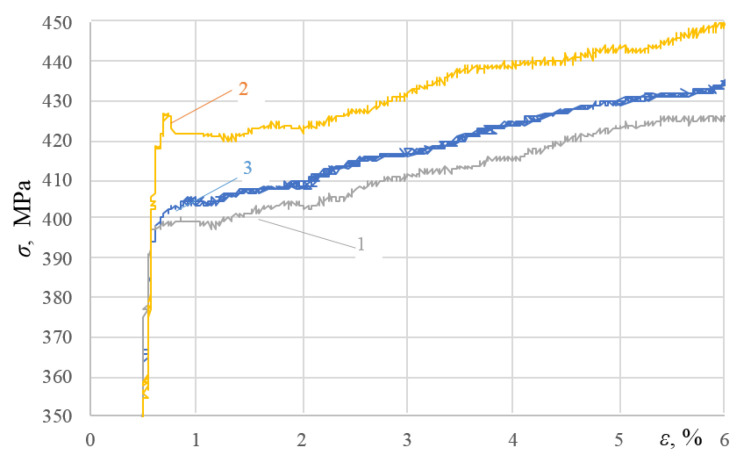
Diagrams of investigated alloys deformation under static tension after DNPs of different intensities: 1—alloy D16ChATW (ε_imp_= 4.26%); 2—alloy 2024-T351 (ε_imp_ = 3.72%); 3— alloy T (ε_imp_ = 4.06%).

**Figure 7:**
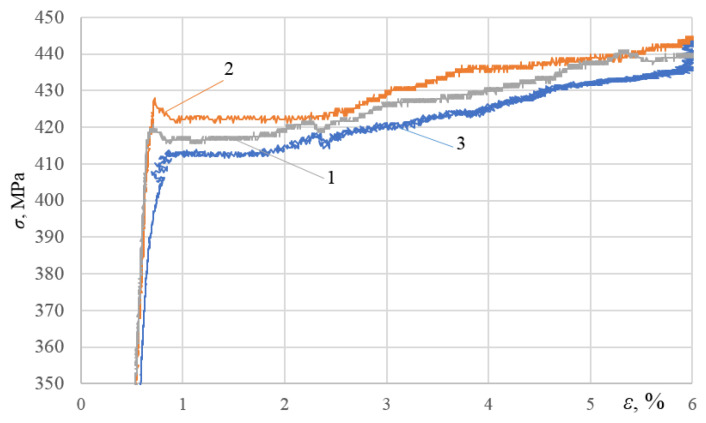
Diagrams of investigated alloys deformation under static tension after DNPs of different intensities: 1—alloy D16ChATW (ε_imp_= 5.40%); 2—alloy 2024-T351 (ε_imp_ = 5.44%); 3—alloy T (ε_imp_ = 5.37%).

To confirm this fact, Figure 6 and Figure 7 present a larger-scale analysis of the initial sections of stress–strain diagrams drawn for the D16ChATW, 2024-T351 and T alloys under repeated static tensioning after DNP (ε_imp_ = 3.72–4.26% and ε_imp_ = 5.37–5.44%).

The analysis of Figure 6 and Figure 7 fully confirms the above conclusions. After DNPs followed by static tensioning, yield sites occurred via different mechanisms in aluminum alloys with significantly different Cu, Mn, and Mg contents. With the mean DNP intensity (ε_imp_ = 4.01%), a sharp yield point was observed in alloy 2024-T351, followed by a yield site (see Figure 6, curve 1). Of the three alloys investigated, the specimen from alloy 2024-T351 was subjected to the lowest DNPs with ε_imp_ = 3.72% (see Figure 6). The yield site also appeared on the specimen from alloy D16ChATW (see Figure 6, curve 2), although its length was shorter than that of the specimen from alloy D16ChATW. However, only rudimentary yield sites were found on the specimen from alloy T (see Figure 6, curve 3). At a much higher DNP intensity, with ε_imp_ = 5.4% (see Figure 7), all the three alloys had yield sites. A sharp yield point was also observed in alloy D16ChATW. Alloy 2024-T351 was characterized by yield sites of the maximum length, while alloy T was characterized by yield sites of the minimum length.

Notably, the mechanical properties of alloys subjected to DNPs have changed upon subsequent static tensioning, as evidenced by the stress–strain diagrams’ elastic sections. This must be taken into account when calculating the strength of load-bearing structures from aluminum alloys that work under impact loads.

### 3.2. Physical Explanation of the Effect Manifested by Yield Sites That Occur in Aluminum Alloys Subjected to DNPs of Different Intensities

From a physical perspective, the effects manifested by yield sites that occur in the alloys subjected to DNPs of different intensities must be directly related to a thin-striped (amorphous) structure formed in the alloys volume [30,31]. We should also note that at room temperature, two main mechanisms of superplastic deformation—diffusion creep and intergranular sliding—were absent in all the studies conducted. In the course of DNPs, the newly created dissipative thin-striped amorphous-like structures [20,21] were seen to cover significant volumes (blocks) of material, the size of which was orders of magnitude greater than that of the original grains.

This is probably the main difference between the superplastic deformation and the phenomenon of yield sites that occur under additional impulse loads applied at room temperature.

**Figure 8:**
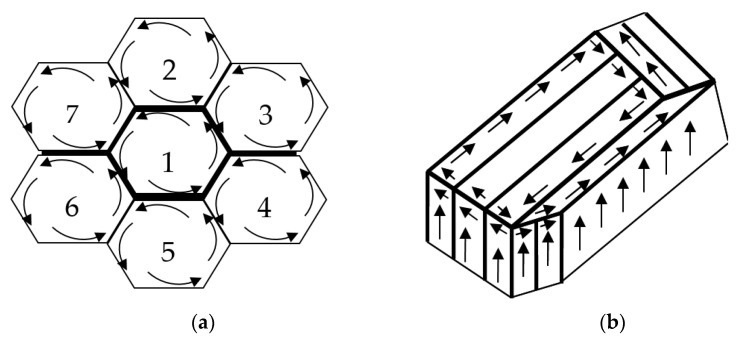
Patterns of superplastic material deformation micro mechanisms of ((**a**), 1–6 grain numbers) and patterns obtained after the additional impulse loads application (**b**).

During superplastic deformation, the main mechanism was grain sliding at the boundaries. In the case investigated, the main mechanism was sliding between blocks of material due to formed liquid-like amorphous structures (hydrodynamic flow channels) [30,31]. As a result, the yield sites formed in the material subjected to additional impulse loads were much smaller in length than the total material deformation under super-plasticity. Superplastic deformation patterns are presented in Figure 8a, and patterns obtained after the application of additional impulse loads are presented in Figure 8b.

Previously, the authors conducted a study dedicated to the statistical width analysis of the dissipative structures’ bands detected and the block structure formed in aluminum alloy D16 and stainless steel and Armco-iron after DNPs [30]. The block structure was shown to be manifested most clearly on Armco-iron [30]. In addition, the density of the nano-created dissipative structure is less than that of the main material. Therefore, numerous micro-extrusions were found on the sheet aluminum alloys surface after the DNPs [28,29].

**Figure 9:**
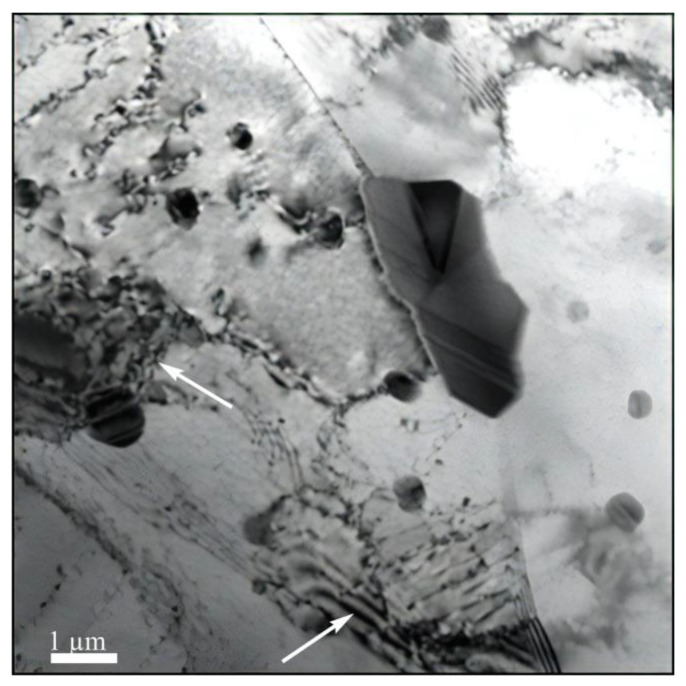
TEM—the alloy structure 2024-T351 after DNP (ε_imp_ = 3.72%); Arrows show block-structure forming zones.

**Figure 10:**
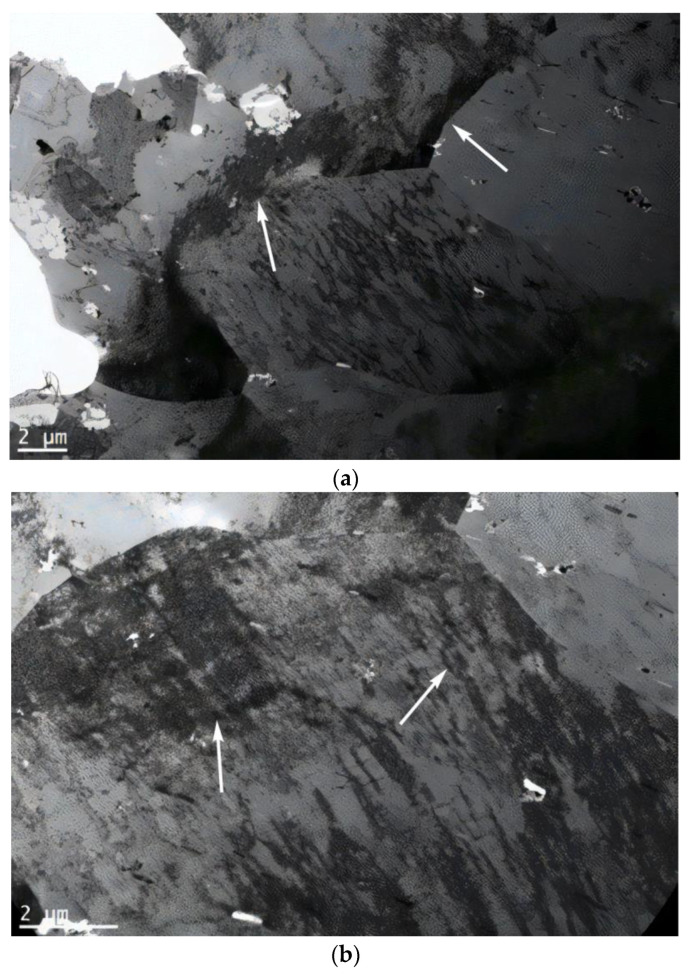
TEM—(**a**,**b**) the structure of alloy D16ChATW after DNP (ε_imp_ = 6.69%); Arrows show the formation of hydrodynamic flow channels and block structure zones.

In this research, the thin foil method (TEM) was used to reveal the thin-striped structures interconnected at different structural levels. In addition, with an increase in the DNP intensity, the number of such bands increases in different directions, thus contributing to the formation of block structures. Figure 9 shows the TEM-structure of alloy 2024-T351 after DNPs with ε_imp_ = 3.72%, and Figure 10 presents the TEM-structures of alloy D16ChATW after the introduction of impulse energy ε_imp_ = 6.69%.

While analyzing Figure 9 and Figure 10, one can notice that at low DNP values, such block structures are present in the alloy only in the rudimentary state (see Figure 9), and that at a much higher DNP intensity the number of block structures increases (see Figure 10).

This suggests that an increase in the DNP intensity causes an increase in the number of dissipative structures in the volume of alloys subjected to DNPs. The proposed alloy deformation physical model (see Figure 8) after DNPs was confirmed experimentally (see Figure 9 and Figure 10).

## 4. Mathematic Process Modelling

The similarities between the superplastic material deformation and material deformation subjected to additional impulse loads make it possible to use the general approach of super-plasticity theory to predict the yield zones length that occur in sheet materials subjected to additional impulse loads. This approach suggests that, within the single curve hypothesis, the stress intensity versus strain rate intensity ratio can be expressed by the ratio using the nonlinear function of the shear viscosity *µ*, which, in general, depends on the chemical *χ* and phase *θ* composition, temperature *T* and strain rate ${\dot{\varepsilon }}_{i}\;$(or applied stress *σ_i_*), and the size of structural components *L*, or Ω and other parameters *p_i_* [34].

(1) $$\sigma_{i}=3\mu \cdot {\dot{\varepsilon }}_{i}$$

(2) $$\mu =f\left(\chi ,\theta ,T,\Omega ,\;L,{\dot{\varepsilon }}_{i},p_{i}\right)$$

Similarly, we introduce the effective shear viscosity concept, which applies when a dissipative thin-band liquid-like structure is formed in the alloy:

(3) $$\mu =\frac{\sigma_{\mathrm{imp}}}{{\dot{\varepsilon }}_{\mathrm{imp}}}$$

where *σ*_imp_ is the dynamic stress, at which additional impulse loads are applied; ${\dot{\varepsilon }}_{\mathrm{imp}}$ is the mean strain rate of alloys when a dissipative liquid-like structure is formed in alloys (%/s) (dynamic jump areas upon DNPs with ε_imp_).

Based on the research findings, Table 3 summarizes the intensities of impulse energy introduced into the alloys, taking into account the ε_imp_ parameter, dynamic stress *σ*_imp_ during DNPs, mean strain rate ε′ during DNPs, effective shear viscosity *μ*, and yield site length ε_y_ for each of the three alloys studied.

**Figure 11:**
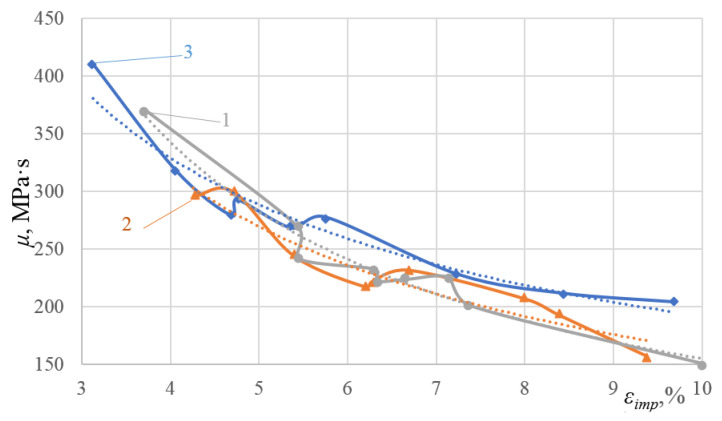
Experimental effective shear viscosity *μ* versus ε_imp_ dependences for aluminum alloys: 1—alloy 2024-T351; 2—alloy D16ChATW; 3—alloy T.

**Figure 12:**
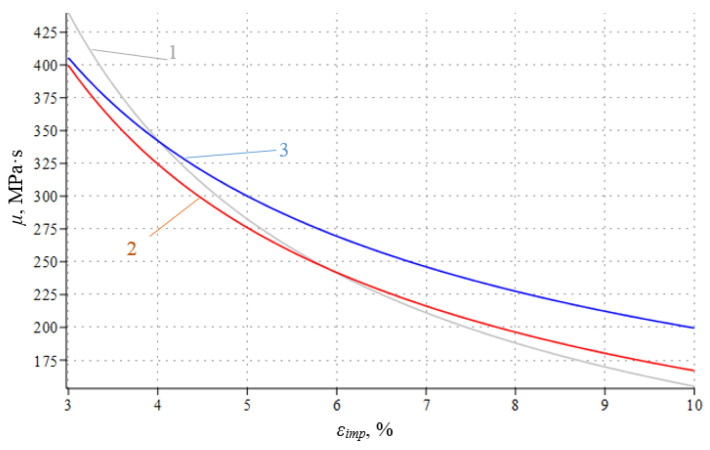
Approximated effective shear viscosity *μ* versus ε_imp_ dependences for aluminum alloys: 1—alloy 2024-T351; 2—alloy D16ChATW; 3—alloy T.

Based on the data obtained, the effective shear viscosity *μ* versus ε_imp_ dependences were plotted and approximated for all the three alloys investigated (Figure 11 and Figure 12).

The corresponding data approximations are shown in Figure 12 below:

For alloy 2024-T351, *μ* = 1138.7 ε_imp_^0.866^, R^2^ = 0.967 (4)

For alloy D16ChATW, *μ* = 866.49 ε_imp_^0.725^, R^2^ = 0.917 (5)

For alloy T, *μ* = 744.32 ε_imp_^0.725^, R^2^ = 0.949 (6)

According to the data analysis presented in Figure 12, alloy 2024-T351 is characterized by the most rapid decrease in the effective shear viscosity *μ* in the given variation range of ε_imp_, that is, 59%. Starting from ε_imp_ = 4%, the effective shear viscosity *μ* for alloys 2024-T351 and D16ChATW is lower than that for alloy T. Moreover, with an increase in ε_imp_, this difference increases. Similarly, the lower the *μ* parameter, the bigger the dissipative structure that appears, while the impulse energy is applied into the alloy. Therefore, based on the data presented in Figure 12, we can accurately predict which alloy will have longer yield sites. As can be seen from Table 3, the yield sites length for certain values of the ε_imp_ parameter can differ up to four-fold, depending on the initial alloy chemical composition.

**Figure 13:**
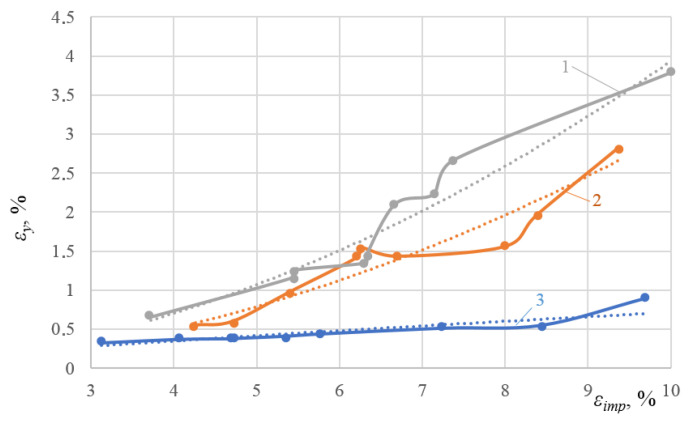
Experimental dependences between DNPs (with the relevant ε_imp_ parameter) and the length of yield sites ε_y_ in alloys: 1—alloy 2024-T361; 2—alloy D16ChATW; 3—alloy T.

Of interest also are the direct dependences between the DNP intensity (with the relevant ε_imp_ parameter) and the yield sites length ε_y_ in alloys. Figure 13 presents the experimental dependences plotted, with the corresponding approximations presented below.

The corresponding data approximations are shown in Figure 13 below:

For alloy 2024-T361, ε_y_ = 0.0523 ε_imp_^1.8765^, R^2^ = 0.937 (7)

For alloy D16ChATW, ε_y_ = 0.0352 ε_imp_^1.9331^, R^2^ = 0.904 (8)

For alloy T, ε_y_ = 0.1193 ε_imp_^0.7781^, R^2^ = 0.809 (9)

## 5. Discussion

**Figure 14:**
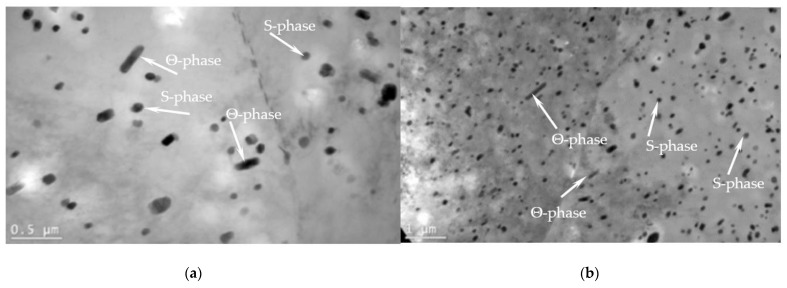
TEM-structures of aluminum alloys in the initial state: (**a**)—alloy 2024-T361; (**b**)—alloy D16ChATW. S-phase and Θ-phase particles are indicated by arrows.

Based on the physical model proposed, which represents alloy deformation after being subjected to DNPs, and the experimental findings (see Table 3 and Figure 11, Figure 12 and Figure 13), we can give an adequate physical explanation for the impact of the alloy’s initial phase composition on the effects manifested by the yield sites that occur in the alloys studied, taking into account an increase in their length. In the initial state, aluminum alloys of complex chemical composition, such as 2024-T351 and D16ChATW, contain a solid aluminum and copper solution strengthened by alloying elements, such as Si, Fe, Mn, Mg, Zn and Ti, and insoluble Al_2_Cu particles (the so-called Θ-phase) and CuAl_2_Mg (the so-called S-phase). The Θ-phase has a tetragonal bcc lattice, a density of 4.345 g/cm^3^, and a melting point of 591 °C. The S-phase has a rhombic face-centered lattice, a density of 3.55 g/cm^3^, and a melting point of 550 °C. The shape of Θ-phase particles represents an asymmetric geometric figure similar to a rectangular parallelepiped with a complex stress field around each particle (Figure 14a). At the same time, S-phase particles have a spherical shape; therefore, the stress field intensity formed by these particles is much lower than that around the Θ-phase particles (Figure 14b).

Both phases have a more complex crystal lattice, as compared to the aluminum lattice (consider the main alloy element and its melting point of 660.3 550 °C). The mismatch between the crystalline aluminum structure and the finely dispersed particles of the Al_2_Cu and CuAl_2_Mg phases in the solid solution causes the alloy to strengthen. In addition, the lower melting point of these phases indicates the possible softening under any energy impact—for instance, during the introduction of impulse energy (DNP). The complex alloys phase composition suggests that there may be several patterns of structural transformations that occur under different mechanical influences. Moreover, these transformations affect the mechanical properties of products made of these alloys in a different way.

The obtained alloy TEM-structure (see Figure 9 and Figure 10) suggests that the more hydrodynamic channels are present in the specimen, the greater is the yield site length. Hydrodynamic channels play an important role in the substance mass transfer when dislocation sliding is inhibited either by the substructure that appeared earlier, or cannot occur with some crystal orientations in general [31,32]. In this case, under the external mechanical field action, so-called structural instability occurs—a complete failure of dislocation boundaries. In dislocation chaos, individual dislocations can move over short distances, creating vacancies. A further interaction between vacancies entails the formation of a structure that can be considered as the nucleus of hydrodynamic channels, that is, a synergistic structure formation (self-organization) [31].

**Figure 15:**
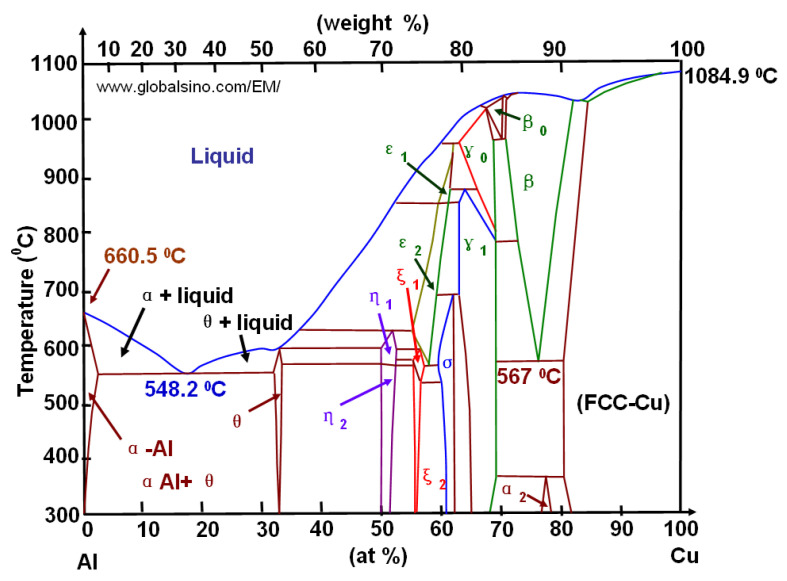
Al-Cu state diagram [35,36].

When we consider the chemical composition of the aluminum alloys studied (Table 2), we can see that these alloys differ significantly, first, in the copper content. At the same time, while analyzing the Al-Cu state diagram (Figure 15), one can see that the content of the θ-phase (Al_2_Cu) increases with an increase in the copper content in the alloy. As shown above, due to its shape, the θ-phase creates micro-stresses in the alloy matrix, which in turn cause the appearance of defects in local areas. These defects are the nuclei of hydrodynamic channels, through which the plastic flow of the substance occurs. Therefore, according to the authors, the main reason for the noticeable effect of the initial phase composition of investigated aluminum alloys on the yield sites appearance of different lengths under DNPs is a significant difference in the θ-phase (Al_2_Cu) content. The conducted research makes it possible to predict the occurrence of yield sites in aluminum alloys under impact-oscillatory loading, primarily based on the copper content in alloys.

## 6. Conclusions

As evidenced by experiments, DNPs cause the formation of yield sites in the elastic part of D16ChATW, 2024-T351 and T alloys upon subsequent static tensioning.

An increase in the Cu content in aluminum alloys causes the appearance of yield sites, the length of which increases. A physical model of this physical phenomenon is proposed, which is based on the similarity with a superplastic deformation. In the case investigated, the main mechanism is slippage between blocks of material due to the liquid-like dissipative amorphous structures (hydrodynamic flow channels) formed during the application of additional impulse loads. The presence of such block structures in the aluminum alloys subjected to DNPs was confirmed by the method of transmission electron microscopy.

A mathematical deformation process model was developed, in which the concept of effective shear viscosity *μ* was introduced as the main characteristic of a dissipative thin-striped liquid-like structure formed in the alloys studied. The *μ* versus ε_imp_ dependences were plotted, and the differences between the yield site lengths were characterized quantitatively.

The alloy initial phase composition impact on the effects manifested by yield sites of varying length that occur in the alloys investigated was physically substantiated and explained. The main reason for the noticeable impact of the alloy’s initial phase composition on the effects manifested by yield sites of different lengths that appear in alloys subjected to DNPs of different intensity is a significant difference in the θ-phase (Al_2_ Cu) content. It is directly related to the initial copper content in alloys. Therefore, the higher the θ-phase content in the alloys, the bigger the dissipative structure in the alloys during the application of additional impulse loads is formed, and the lower is the effective shear viscosity *μ* in the alloys.

## Figures and Tables

**Table 1:** Chemical composition of aluminum alloys, %.

Alloys	Si	Fe	Cu	Mn	Mg	Cr	Zn	Ti
D16ChATW	0.18	4.4	0.63	1.4	0.01	0.01	0.07	-
2024-T351	0.05	0.13	4.7	0.70	1.5	0.01	0.02	0.04
T	0.08	0.255	0.825	0.024	0.03	0.002	0.012	0.018

**Table 2:** Mechanical properties of aluminum alloys D16ChATW.

Alloys	*σ*_ys_, MPa	*σ*_us_, MPa	δ, %
D16ChATW	322	448	25.2
2024-T351	372	462	20.5
T	330	450	21.1

**Table 3:** The main mechanical parameters that characterize the DNP process and the corresponding lengths of the yield sites that occur in the alloys investigated.

Alloy	Number of Specimens	ε_imp_, %	*σ*_imp_, MPa	${\dot{\varepsilon }}_{\mathrm{imp}}$, s^−1^	*μ*, MPa·s	Yield Site, ε_y,_ %
T	1	3.13	300.76	0.73	412.0	0.33
	2	4.06	295.74	0.93	318.0	0.37
	3	4.69	306.36	1.05	291.77	0.38
	4	4.75	316.02	1.13	279.66	0.38
	5	5.37	344.40	1.29	266.98	0.41
	6	5.75	354.34	1.28	276.83	0.44
	7	7.25	343.81	1.51	227.69	0.52
	8	8.44	380.94	1.80	211.63	0.55
	9	9.69	393.28	1.93	203.77	0.90
D16ChATW	1	4.26	374.75	1.27	295.08	0.55
	2	4.71	401.54	1.34	299.66	0.60
	3	5.40	392.70	1.61	243.91	0.97
	4	6.21	402.35	1.85	217.49	1.41
	5	6.27	410.65	1.85	221.97	1.54
	6	6.69	413.75	1.78	232.44	1.43
	7	8.0	404.74	1.95	207.56	1.56
	8	8.39	396.46	2.06	192.46	1.96
	9	9.38	395.94	2.52	157.12	2.83
2024-T351	1	3.72	398.15	1.08	368.66	0.66
	2	5.43	437.30	1.62	269.94	1.16
	3	5.44	398.20	1.65	241.33	1.26
	4	6.31	427.38	1.84	232.27	1.35
	5	6.32	417.18	1.88	221.90	1.45
	6	6.67	422.86	1.89	223.74	2.11
	7	7.14	425.19	1.89	224.97	2.23
	8	7.38	428.06	2.13	200.97	2.67
	9	10.0	413.06	2.74	150.75	3.79

## Data Availability

The data presented in this study are available upon request from the corresponding author.
